# Clinical Characteristics of Gliosarcoma and Outcomes From Standardized Treatment Relative to Conventional Glioblastoma

**DOI:** 10.3389/fonc.2019.01425

**Published:** 2019-12-17

**Authors:** Simone Frandsen, Helle Broholm, Vibeke Andrée Larsen, Kirsten Grunnet, Søren Møller, Hans Skovgaard Poulsen, Signe Regner Michaelsen

**Affiliations:** ^1^Department of Radiation Biology, Rigshospitalet, Copenhagen, Denmark; ^2^Department of Pathology, Rigshospitalet, Copenhagen, Denmark; ^3^Department of Radiology, Rigshospitalet, Copenhagen, Denmark; ^4^Department of Oncology, Rigshospitalet, Copenhagen, Denmark; ^5^Biotech Research and Innovation Centre (BRIC), Faculty of Health and Medical Sciences, University of Copenhagen, Copenhagen, Denmark

**Keywords:** gliosarcoma, glioblastoma, radiation, temozolomide, survival, tumor location

## Abstract

**Background:** Gliosarcoma (GS) is a rare histopathologic variant of glioblastoma (GBM) characterized by a biphasic growth pattern consisting of both glial and sarcomatous components. Reports regarding its relative prognosis compared to conventional GBM are conflicting and although GS is treated as conventional GBM, supporting evidence is lacking. The aim of this study was to characterize demographic trends, clinical outcomes and prognostic variables of GS patients receiving standardized therapy and compare these to conventional GBM.

**Methods:** Six hundred and eighty GBM patients, treated with maximal safe resection followed by radiotherapy with concomitant and adjuvant temozolomide at a single institution, were retrospectively reevaluated by reviewing histopathological records and tumor tissue for identification of GS patients. Clinico-pathological- and tumor growth characteristics were obtained via assessment of medical records and imaging analysis. Kaplan-Meier survival estimates were compared with log-rank testing, while Cox-regression modeling was tested for prognostic factors in GS patients.

**Results:** The cohort included 26 primary gliosarcoma (PGS) patients (3.8%) and 7 secondary gliosarcoma (SGS) patients (1.0%). Compared to conventional GBM tumors, PGS tumors were significantly more often MGMT-unmethylated (73.9%) and located in the temporal lobe (57.7%). GS tumors often presented dural contact, while extracranial metastasis was only found in 1 patient. No significant differences were found between PGS and conventional GBM in progression-free-survival (6.8 and 7.6 months, respectively, *p* = 0.105) and in overall survival (13.4 and 15.7 months, respectively, *p* = 0.201). Survival following recurrence was not significantly different between PGS, SGS, and GBM. Temporal tumor location and MGMT status were found associated with PGS survival (*p* = 0.036 and *p* = 0.022, respectively).

**Conclusion:** Despite histopathological and location difference between GS and GBM tumors, the patients present similar survival outcome from standardized treatment. These findings support continued practice of radiation and temozolomide for GS patients.

## Introduction

Glioblastoma (GBM, WHO grade IV glioma) is the most common and aggressive primary brain tumor in adults with a median overall survival (OS) of around 15 months (1). Gliosarcoma (GS) is a rare histopathological variant of isocitrate dehydrogenase (IDH)-wildtype GBM and accounts for ~2% of all GBM (1–5), although frequencies up to 8% have been reported (6, 7). Histologically, GS tumors are characterized by a biphasic growth pattern consisting of both glial components and areas of sarcomatous, mesenchymal differentiation often resembling fibrosarcoma (7). The mesenchymal components may also comprise chondral (8, 9), osteoid (9–11), osteochondral (12, 13), myomatous (14, 15), and/or lipomatous (16) elements. The pathogenesis of GS remains unknown, but findings of common genetic alterations in both the gliomatous and sarcomatous components support the hypothesis that GS tumors are of monoclonal origin (17–20).

GS are termed primary gliosarcoma (PGS) if they arise *de novo* without any prior GBM diagnosis, whereas GS occurring after treatment of conventional GBM are termed secondary gliosarcoma (SGS). SGS are distinguished from radiation therapy (RT)-induced GS, which arise after intracranial RT in patients without any prior presence of GBM (21–24). GS most often affects adults in the fifth to seventh decade of life, with a male predominance, and has a temporal lobe predilection (4–6, 25–27). On imaging GS lesions typically present as a well-demarcated supratentorial mass often peripherally located and abutting dura (26–31). While these imaging features are more likely to occur in GS compared to conventional GBM, it is still not possible to diagnose GS by imaging alone (27, 30). The growth pattern of GS tumors may differ from that of conventional GBM, as extracranial (EC) metastasis has been reported in up to 11% of GS (32), which is far more than among conventional GBM patients, with <2% of cases metastasizing (33, 34). Additionally, cases of skull base invasion and EC extension have been described (30, 35, 36).

While an exceptional poor prognosis for GS has been reported (5, 29), several studies showed no significant differences in outcome between GS and conventional GBM (3, 25, 37). GS patients are typically managed as conventional GBM in accordance with the Stupp's regimen of trimodality therapy including maximal safe resection, RT with concurrent and adjuvant temozolomide (TMZ) based chemotherapy (38). However, solid supporting evidence for this strategy is lacking. Only few studies have conducted regular comparisons of standardized concomitant RT and chemotherapy in GS vs. conventional GBM patients and these are disposed to uncertainties like insufficient patient number or lack of information on precise therapeutic intervention (25, 29, 37, 39). For GBM identified prognostic factors include patient age, performance status (PS), extent of resection (EoR), corticosteroid use at start of treatment and methylation of the gene promoter of O^6^-methylguanine-DNA-methyltransferase (MGMT) (40, 41), a DNA repair protein inhibiting the effect of TMZ (42). Patient age and EoR were also found prognostic among GS patients in a large registry study (37) but prognostic influence in GS of other variables such as MGMT promoter methylation remains uncertain (30, 37, 43).

In this report, we retrospectively reviewed a series of GS patients to characterize demographic trends, prognostic variables and clinical outcomes. To evaluate the current clinical management of GS we compared survival after standardized treatment for PGS patients relative to conventional GBM patients.

## Methods

### Patients

**Figure 1:**
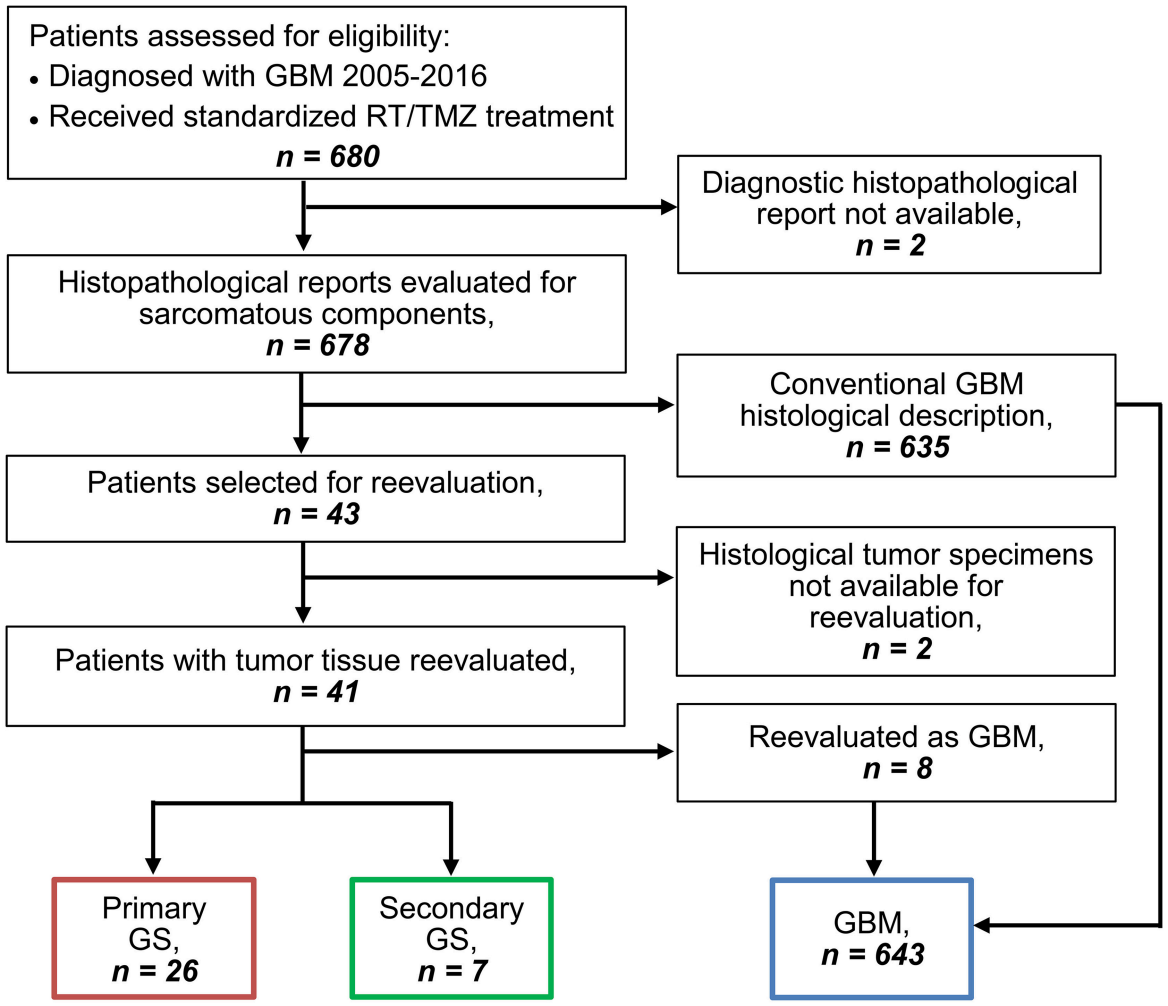
REMARK diagram for identification of GS and GBM patients.

Six hundred and eighty patients were from January 2005 to December 2016 diagnosed with GBM and treated according to Stupp's regimen at Rigshospitalet, Copenhagen, Denmark. All patients were retrospectively evaluated for study eligibility by reviewing the histopathological reports from diagnosis of tumor tissue from both primary and later GBM surgeries. Two patients without available histopathological reports were excluded and reports from the remaining 678 patients were reviewed for description of a sarcomatous appearance and/or component of the tumor. Forty-three patients were selected for histological reevaluation based on the reports, of which two had to be excluded due to lacking histopathological specimens. The histopathological specimens of the remaining 41 patients' tumor tissue were evaluated for presence of a sarcomatous component. The GS diagnosis was made based on a biphasic growth pattern on hematoxylin- and eosin (HE)-staining as well as glial fibrillary acidic protein (GFAP)-staining demonstrating GFAP-positive gliomatous components and GFAP-negative sarcomatous components containing neoplastic spindle-shaped cells. Furthermore, the diagnosis was supported via evaluation for collagen richness in the sarcomatous components by Van Gieson staining. Evaluation was performed independently by two assessors (one being a trained neuropathologist) and consensus was followingly achieved. Both assessors were blinded to clinical data. The GS tumors presented a highly heterogeneous histological pattern of sarcomatous components. Of this reason there was no cut-off to the extent of sarcomatous components of the tumor, all tumors containing a sarcomatous component were considered as GS. Primary gliosarcomas (PGS) were defined as *de novo* tumors in patients with no prior history of GBM. Secondary gliosarcomas (SGS) were defined based on histopathological diagnosis of GS at reresection following previous diagnosis and treatment of conventional GBM. Patient selection for this study is demonstrated in the REMARK diagram in Figure 1.

### Treatment

All patients, irrespectively of age, received first-line Stupp's regimen (i.e., TMZ 75 mg/m^2^/day plus RT at a dose of 60 Gy to the planning target volume in 30 fractions with 5 fractions/week, followed by up to 6 courses of adjuvant TMZ therapy each consisting of 150–200 mg/m^2^/day TMZ for 5 days followed by 23 days without therapy). More details on administration has previously been described (40). After progression on primary therapy, 250 patients underwent reresection and 280 received bevacizumab in most cases given together with irinotecan or lomustine (CCNU) dependent on the local guidelines at the time (44). Selected patients further received different types of experimental treatment either before or after bevacizumab recurrence therapy.

### Assessment of Patient and Tumor Growth Characteristics

Baseline demographic information and treatment-related variables including age, sex, PS (ECOG score), anatomic tumor localization, multifocality, tumor size, EoR, use of corticosteroids, site of recurrence and re-resection were collected via medical records when available. For the PGS patients, when available, preoperative, 72 h post-operative and later magnetic resonance imaging (MRI) were re-assessed by a trained neuroradiologist regarding anatomical localization, multifocality, contact to dura, EoR and metastatic spread. EoR was defined as subtotal if residual tumor tissue was ≥ 1 × 1 × 1 cm and site of recurrence was defined as distant if more than 1 cm from primary tumor location. If not available on imaging, the medical records, including histopathological reports, were searched for information on distant intracranial recurrence, EC extension and distant metastases. Status for mutation of IDH1 and promoter methylation of MGMT were obtained during routine tissue examination from primary GBM/GS surgery by varying detection methods dependent on the time of analysis. IDH1 was either examined by immunohistochemistry (IHC) using anti-IDH1 R132H antibody (clone H09, Dianova, 1:700 dilution) or by Multiplex Ligation-dependent Probe Amplification (MLPA), using the SALSA MLPA kit P088 (MRC Holland, Amsterdam, the Netherlands). MGMT promoter methylation was from 2014 analyzed by pyrosequencing using the Therascreen MGMT Pyro kit (Qiagen) considering a mean methylation above 10% as positive. Until then it was determined indirectly by a non-standardized IHC method using anti-MGMT antibody (MAB16200, Millipore, 1:200). In previous publication we found this indirect method for estimation of MGMT promoter methylation to be strongly associated with results obtained by pyrosequencing, although it is less sensitive and underestimate the number of unmethylated samples (45).

### Survival Endpoints and Statistical Evaluation

Patient survival was calculated as follows: Progression-free-survival (PFS) was defined as the time from initial GBM/GS diagnosis until first recurrence with radiological or clinical progression or alternative death without prior disease recurrence. OS was calculated as the time from diagnosis until date of death and survival from recurrence as the time from first recurrence until death. OS for SGS patients was calculated from the date of histopathologic confirmed GS diagnose until time of death. Time to SGS was calculated from date of initial GBM diagnosis until date of recurrence resection with histopathologically confirmation of GS.

Survival probabilities were estimated using Kaplan-Meier method and survival differences between the histopathological groups evaluated with Mantel-Cox log rank test. Comparison of clinical characteristics for PGS and GBM cohorts were done using Fisher's exact test and Mann–Whitney *U*-Test. Univariate analyses were conducted by Cox proportional hazards modeling also estimating hazard ratios (HR) with a 95% confidence interval (CI). *P* ≤ 0.05 were considered significant. Calculations were performed using SPSS (v22.0, IBM Corp., Armonk, NY).

## Results

### Characteristics of PGS Patients Compared to Conventional GBM

Among 680 GBM patients diagnosed from 2005 to 2016 and treated according to Stupp's regimen we identified 643 conventional GBM patients, 26 PGS patients (3.8% of all GBM) and 7 SGS patients (1.0% of all GBM), excluded patients = 4 (REMARK diagram, Figure 1). Comparison of clinico-pathological characteristics of PGS and conventional GBM (Table 1) revealed no significant differences in regard to median patient age at diagnosis (59.7 vs. 60.3, *p* = 0.804), sex (73.1 vs. 65% males, *p* = 0.529), pretreatment PS (61.5 vs. 61.9% with PS = 0, *p* = 1.000) or corticosteroid use at treatment start (76.9 vs. 61.1%, *p* = 0.148). Additionally, no differences were found regarding multifocal disease (multifocal in 19.2 vs. 12%, *p* = 0.353), median tumor size (1,610 vs. 1,600 mm^2^, *p* = 0.348) or hemisphere laterality (right side in 65.4 vs. 47.1%, *p* = 0.119). PGS tumors were most often located in the temporal lobe (57.7%) followed by multilobar location (19.2%), frontal lobe (15.4%), occipital lobe (3.85%), and parietal lobe (3.85%). Compared to conventional GBM, PGS tumors were significantly more frequent located to the temporal lobe (27.5 vs. 57.7%, *p* = 0.002). At disease recurrence, registered in all but one of the PGS patients, no difference was seen regarding having a distant or local recurrence site in PGS compared to conventional GBM (*p* = 0.608) (Supplementary Table 1).

**Table 1:** Patient characteristics of conventional GBM and PGS.

**Variable**	**GBM** ** (*n* = 643)**	**PGS** ** (*n* = 26)**	***P*-value** ** (PGS vs. GBM)**
Age at diagnosis (years), median (range)	60.3 (17.1–79.6)	59.7 (25.6*-*76.8)	0.804
Sex, *n* (%)			0.529
Male	418 (65.0)	19 (73.1)	
Female	225 (35.0)	7 (26.9)	
PS, *n* (%)			1.000
0	393 (61.9)	16 (61.5)	
1–2	242 (38.1)	10 (38.5)	
Missing	8	0	
Corticosteroid use^*^, *n* (%)			0.148
No	248 (38.9)	6 (23.1)	
Yes	389 (61.1)	20 (76.9)	
Missing	6	0	
Location, lobe^**^, *n* (%)			
Frontal	132 (20.6)	4 (15.4)	0.627
Temporal	176 (27.5)	15 (57.7)	0.002
Parietal	74 (11.5)	1 (3.85)	0.345
Occipital	46 (7.2)	1 (3.85)	1.000
Multilobar	163 (25.4)	5 (19.2)	0.646
Profound	17 (2.7)	0 (0.0)	
Other	33 (5.1)	0 (0.0)	
Missing	2	0	
Location, hemisphere, *n* (%)			0.119
Right	301 (47.1)	17 (65.4)	
Left	293 (45.9)	7 (26.9)	
Profound/bilateral	45 (7.0)	2 (7.7)	
Missing	4	0	
Multifocal disease, *n* (%)			0.353
No	564 (88)	21 (80.8)	
Yes	77 (12)	5 (19.2)	
Missing	2	0	
Tumor size (mm^2^), median (range)	1600.0 (25–6,150)	1610.0 (625–5,046)	0.348
Missing	328	9	
EoR, primary surgery, *n* (%)			0.004
Biopsy	100 (15.7)	0 (0.0)	
Subtotal resection	237 (37.2)	17 (65.4)	
Gross total resection	300 (47.1)	9 (34.6)	
Missing	6	0	
Adjuvant TMZ therapy, *n* (%)			0.293
≥6 cycles	227 (35.4)	6 (23.1)	
<6 cycles	415 (64.6)	20 (76.9)	
Missing	1	0	
MGMT status^***^, *n* (%)			0.009
Methylated	292 (54.6)	6 (26.1)	
Unmethylated	243 (45.4)	17 (73.9)	
Missing	108	3	
IDH1 status, *n* (%)			0.614
Wild-type	372 (95.6)	22 (100.0)	
Mutated	17 (4.4)	0 (0.0)	
Missing	254	4	

*Statistical tests: Mann-Whitney U-test (Age, tumor size); Fisher's exact test (Sex, PS, Corticosteroid use, Location, Multifocal disease, EoR, adjuvant therapy, MGMT status, IDH1 status). GBM, glioblastoma; PGS, primary gliosarcoma; PS, performance status; EoR, extent of resection; TMZ, temozolomide; MGMT, O6-methylguanine-DNA-methyltransferase; IDH1, isocitrate dehydrogenase 1*.

* *Prednisolone ≥ 10mg*.

** *Tumor involving more than one lobe*.

*** *Estimated directly by pyrosequencing or indirectly by IHC, details is shown in Supplementary Table 2*.

In contrast to conventional GBM, all PGS were IDH1 wildtype (100%), but statistical analysis did not reveal any significant difference (*p* = 0.614). Contrary, there was a significantly lower frequency of MGMT promoter methylation in PGS when compared to conventional GBM (26.1 vs. 54.6%, *p* = 0.009) (Table 1 and Supplementary Table 2).

EoR at primary surgery was recorded in all 26 PGS patients who underwent either gross total resection (34.6%) or subtotal resection (65.4%), a distribution that turned out significantly different from conventional GBM (*p* = 0.004) (Table 1). Otherwise treatment of PGS did not differ significantly from conventional GBM, with 6 PGS patients receiving a minimum of 6 cycles adjuvant TMZ during first-line treatment (*p* = 0.293), 12 PGS patients undergoing reresection (*p* = 0.408) and 10 PGS patients receiving systemic bevacizumab salvage therapy (*p* = 0.840), given as bevacizumab and irinotecan combined (*n* = 6), or bevacizumab and CCNU combined (*n* = 4) (Supplementary Table 1). Other systemic salvage therapies for the PGS patients included CCNU monotherapy (*n* = 4), selinexor (*n* = 2), palliative TMZ (*n* = 1), and nintedanib (*n* = 1). One PGS patient developed several subcutaneous metastasis (noduli) in the neck after 5 series of adjuvant TMZ and had a subtotal removal of the tumors due to muscle infiltration.

### Imaging Characteristics of PGS Patients

Preoperative MRI were available in 23 PGS patients, while 15 PGS patients had available 72 h post-operative MRI (Table 2). The tumors of 15 patients (58%) presented with dural contact at time of diagnosis while two additional patients had a tumor with dural contact at time of progression (case 9 and 21). A total of 6 PGS patients had distant intracranial recurrence with the tumor(s) located elsewhere than the primary tumor. Two patients (case 11 and 13) had at recurrence a tumor growing through the previous operation channel into the subcutaneous layer of the scalp. Only one patient (case 24) had EC metastasis in the neck. The radiological evaluation did not support an association between tumor location and EoR, multifocality, dural contact, intracranial spread, or EC growth, respectively.

**Table 2:** Imaging characteristics of PGS tumors.

**Case**	**Sex**	**Age range, debut**	**Location**	**Multifocal, debut**	**EoR, primary surgery**	**Dural contact**	**Distant intracranial recurrence**	**EC extension**	**Distant metastasis**
1	M	50–59	L Temporal	÷	STR^*^	Unk	÷^a^	÷^a^	÷^a^
2	F	50–59	L Temporal	÷	STR^*^	+	÷	÷	÷
3	F	60–69	R Frontoparietal	÷	GTR^*^	Unk	÷^a^	÷^a^	÷^a^
4	M	40–49	L Frontotemporal	÷	STR^*^	+	÷	÷	÷
5	M	70–79	R Temporal	÷	STR^*^	÷^b^	÷	÷	÷
6	M	60–69	R Frontoparietal	÷	GTR	÷	÷	÷	÷
7	M	60–69	Bilat Frontal	+	STR	+	÷	÷	÷
8	M	60–69	R Temporooccipital	+	STR	+	÷	÷	÷
9	M	50–59	L Temporal	÷	STR	+ (progression)	÷	÷	÷
10	F	50–59	L Frontal	÷	STR	+	÷	÷	÷
11	M	50–59	R Temporal	÷	GTR	+	÷	+ (subcutis)	÷
12	M	40–49	R Temporal	÷	STR^*^	+	÷	÷	÷
13	F	40–49	R Temporal	÷	STR	+	÷	+ (subcutis)	÷
14	M	60–69	R Temporal	+	STR	+	÷	÷	÷
15	M	50–59	R Temporal	÷	GTR	+	+ (R frontal, cerebellar)	÷	÷
16	F	70–79	R Occipital	÷	STR	+	÷	÷	÷
17	M	70–79	R Temporal	÷	GTR	+	+ (L frontal)	÷	÷
18	M	60–69	L Temporal	÷	STR	+	+ (L frontotemporal)	÷	÷
19	M	50–59	R Temporal and occipital	+	STR^*^	+	÷	÷	÷
20	M	40–49	R Temporal	+	STR^*^	+	+ (L frontal, temporal, occipital)	÷	÷
21	M	50–59	R Temporal	÷	STR^*^	+ (progression)	÷	÷	÷
22	M	20–29	Bilat Frontal	÷	STR	÷	+ (fossa posterior, 4. ventricle)	÷	÷
23	M	70–79	R Frontal	÷	GTR	÷	÷	÷	÷
24	M	60–69	L Parietal	÷	GTR^*^	÷	÷	÷	+ (neck)
25	F	60–69	R Temporoparietal	÷	GTR	÷	+ (L cerebellar)	÷	÷
26	F	70–79	R Temporal	÷	GTR^*^	÷	÷	÷	÷

*M, male; F, female; L, left; R, right; EoR, extent of resection; STR, subtotal resection; GTR, gross total resection; Unk, unknown; EC extracranial*.

* *EoR based on surgical records*.

a *Information based on medical records*.

b *Information based on imaging descriptions*.

### Characteristics of SGS Patients

Characteristics of the seven SGS patients are displayed in Table 3. The median age for SGS patients at diagnosis for conventional GBM was 55 years (range 23–65 years) and SGS developed in patients of both sexes and with varying PS. These tumors were both MGMT methylated and unmethylated tumors and presenting various tumor location, although involvement of the frontal and parietal lobes was dominating. All SGS patients were diagnosed at first reresection for conventional GBM, while their diagnostic tumor tissue from their first surgery did not show any sarcomatous components, but conventional GBM histology. One patient did not receive any salvage therapy for SGS and three patients underwent reresection for recurrent SGS. Salvage chemotherapy included bevacizumab and irinotecan (*n* = 3), bevacizumab and CCNU (*n* = 1) or cilengetide (*n* = 1). Two SGS patients (case 2 and 3) had at time of progression a tumor growing through the operation channel involving the scalp and subcutaneous layer, one of these patients (case 3) received salvage RT.

**Table 3:** Characteristics of SGS patients.

**Case**	**Sex**	**Age range, debut**	**MGMT status**	**IDH status**	**PS at debut**	**Location of primary GBM tumor**	**EOR, GBM**	**GBM treatment prior to SGS surgery**	**Time to SGS (months)**	**Location of SGS**	**EOR, SGS**	**SGS treatment**	**OS from SGS**	**OS from initial GBM diagnosis (months)**
1	M	50–59	÷	WT	1	Frontal	STR	RT + TMZ (6 cycles adj.)	13.2	Frontal	GTR	Re-OP for SGS relapse	9.0	22.3
2	F	60–69	÷	WT	0	Parieto-occipital	GTR	RT + TMZ (6 cycles adj.)	10.2	Parietal	STR	2 cycles Bev/CCNU,Re-OP for SGS relapse	8.0	18.3
3	M	60–69	÷	WT	1	Frontal	STR	RT + TMZ (2 cycles adj.)	5.4	Frontal	STR	4 cycles Bev/Iri, salvage RT of subcutaneous tumor	6.6	12.0
4	M	50–59	+	WT	0	Multifocal parietal	Biopsy	RT + TMZ (0 cycles adj.), followed by OP	19.3	Fronto-parietal	STR	None	1.2	20.5
5	M	50–59	÷	Unk	2	Fronto-parietal	STR	RT + TMZ (2 cycles adj.)	5.7	Fronto-parietal	STR	4 cycles Bev/Iri	6.7	12.2
6	F	60–69	+	WT	0	Temporal	STR	RT + TMZ (6 cycles adj.)	10.9	Temporal	STR	2 cycles Bev/Iri, Re-OP for SGS relapse	7.7	18.6
7	F	20–29	Unk	Unk	0	Fronto-parietal	GTR	RT + TMZ (2 cycles adj.), followed by 2 cycles Ce/Bev/Iri, 10 cycles Bev/Iri, 2 cycles Bev/Tor	19.6	Parietal	STR	Experimental treatment with Cilengetide (3 treatments)	2.2	21.8

*SGS, secondary gliosarcoma; M, male; F, female; GBM, glioblastoma; MGMT, O6-methylguanine-DNA-methyltransferase; WT, wildtype; PS, performance status; EoR, extent of resection; STR, subtotal resection; GTR, gross total resection; RT, radiation; TMZ, temozolomide; OP, operation; Ce, cetuximab; Bev, bevacizumab; Iri, irinotecan; Tor, torisel*.

### Patient Outcomes and Prognostic Variables

**Figure 2:**
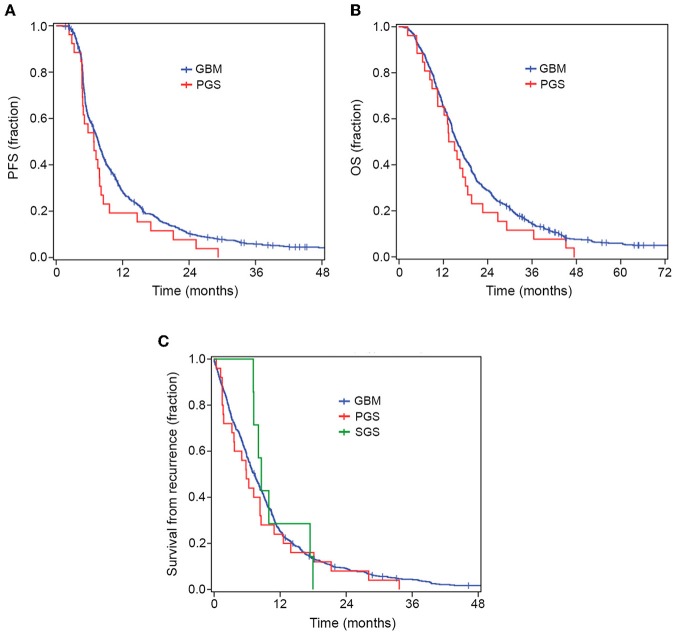
Kaplan–Meier curves for OS, PFS and survival from recurrence. There was no significant difference in PFS **(A)** or OS **(B)** between PGS and conventional GBM. When comparing all GBM, PGS and SGS groups there was no significant difference in survival from recurrence **(C)**.

All PGS and SGS patients had died before the time of analyses, while 32 conventional GBM patients were still alive with a follow-up time of 29–167 months. Figure 2 and Supplementary Table 3 show survival data for the patient groups. PGS patients had a median PFS of 6.8 months (*range* 2.3–29.2) and conventional GBM patients 7.6 months (*range* 1.1–167.7). Median OS for PGS patients was 13.4 months (*range* 2.3–47.4) while conventional GBM had a median OS of 15.7 months (*range* 1.1–167.7). We found no significant difference in median PFS (*p* = 0.105) or median OS (*p* = 0.201) between the groups.

The median time from diagnosis of conventional GBM to diagnosis of SGS was 10.9 months (*range* 5.4–19.6 months). Median survival following SGS diagnosis was 6.6 months (*range* 1.2–9.0 months), while median OS for SGS patients, from diagnosis of conventional GBM to date of death, was 18.6 months (*range* 12.0–22.3 months) (Table 3). When comparing all three groups, i.e., conventional GBM, PGS and SGS, there was no significant difference in median survival from first recurrence (*median* 7.4, 5.8, and 8.6 months, respectively, *p* = 0.694) (Supplementary Table 3).

To investigate the influence of clinico-pathological variables on PFS and OS in PGS patients, we performed univariate analysis. Factors tested were those found significantly different between PGS and conventional GBM patients (temporal tumor location, MGMT promoter methylation and EoR at primary surgery) together with other variables previously demonstrated prognostic for GBM (age, PS, corticosteroid use) (Supplementary Table 4). Factors significantly associated with OS included temporal tumor location (temporal vs. other, *HR*: 0.41, *p* = 0.036) and MGMT promoter methylation (methylated vs. unmethylated, *HR*: 0.17, *p* = 0.022). For PFS MGMT promoter methylation was the solitary factor showing a significant association (*HR*: 0.26, *p* = 0.035).

## Discussion

Although GS has been known for over a century (46) and today is recognized as a distinct histopathologic entity, the best practice for GS is associated with high uncertainty. The influence of the sarcomatous abundance in GS tumors has previously been discussed (47, 48) but recent studies show no association between the extent of sarcomatous components and median OS of GS patients (30, 48). Currently there are no guidelines regarding a cutoff for the extent of sarcomatous component when diagnosing GS tumors (1) and consequently, all patients with a sarcomatous component of the tumor in this study were considered GS, regardless of the sarcomatous quantity. Supporting selection of this cutoff is our finding of an incidence of PGS patients of 3.8% of all GBM, consistent with earlier estimates of 1.8–8% (2, 5, 7). Our PGS cohort was also demographically and clinically comparable with previous described cases of GS (37, 39, 49), patients were predominantly middle-aged men (M:F ratio 19:7, median age 59.7) with a tumor located in the temporal lobe (57.7%). Also, in concordance with GS being a variant of GBM, IDH-wildtype, and prior reported genetic alterations (50, 51) all PGS tumors were IDH1-wildtype.

It was recently proposed that the GS diagnosis could be estimated by preoperatively imaging analysis (27), but this is conflicted by a study finding a slightly larger area of edema to be the only distinct feature of GS compared to conventional GBM tumors upon evaluation of the radiological VASARI feature set (51). Radiological analysis of our PGS patients revealed that 58% had a peripheral tumor abutting dura, consistent with previous reported imaging characteristics (28–31). Skull base invasion and EC extension has been reported a rarity in GS patients (30, 36) and we too found only two PGS patients with EC extension. In contrast to reports of an EC metastasis rate in GS as high as 11% (32), only one PGS patient in our cohort (3.8%) presented with EC metastases and none of the SGS patients developed metastasis. Nevertheless, this low incidence of metastatic spread from GS in our cohort is also supported by other studies, with Cachia et al. (49) reporting that one PGS patient (4.2%) and one SGS patient (10%) developed extra-axial metastasis and two other studies (30, 39) with no incidence of distant metastasis in cohorts of 15 and 22 PGS patients, respectively. Of notice, while we found having a temporal tumor location being associated with improved OS among PGS patients, the radiological exploration did not reveal this to be specially associated with specific growth patterns or EoR.

While MGMT promoter methylation is a known prognostic factor for outcome among GBM patients specifically associated with TMZ efficacy (41), its influence remains uncertain in PGS patients with varying reports on frequency of MGMT promoter methylation and its association with OS. In a study of 12 GS patients with 6 having MGMT promoter methylation and 9 receiving TMZ, Kang et al. (43) found MGMT promoter methylation to be a positive prognostic factor for OS and PFS. Also, in a large registry-based study by Frandsen et al. (37) patients with MGMT promoter methylation trended toward a better median survival when compared to GS patients with unmethylated MGMT promoter. However, the number of patients receiving TMZ was unknown as the specific type of chemotherapy administrated to patients was not registered (37). In contrast, Singh et al. (48) detected MGMT promoter methylation in 5 of 16 GS patients treated with TMZ but found no association to median survival. In this study we found that PGS patients had a significantly lower frequency of MGMT promotor methylation (26.1%) when compared to conventional GBM (54.6%). Still MGMT status was significantly associated with both PFS and OS for TMZ treated PGS patients in univariate analysis. That our level for conventional GBM patients having MGMT promoter methylation was slightly higher than previously reported (41, 52) must be accredited to the combined use of pyrosequencing and an indirect IHC detection method, of which the latter has been found to slightly overestimate number of methylated tumors compared to standard pyrosequencing (45).

The therapeutic effect of TMZ among GS patients has been widely discussed and results from trimodality treatment with TMZ-based chemotherapy are conflicting (53–57). Two previous studies found no significant improvement in OS among GS patients receiving RT with concurrent and adjuvant TMZ compared to RT alone or combined with other chemotherapy (54, 57). In contrast, one study reported that concurrent and adjuvant TMZ improved OS at 24 months from 10.2 to 20% (53) and Adeberg et al. (55) found concomitant TMZ to be significantly associated with increased OS (*p* = 0.01) when compared to RT alone (55). Even with trimodality treatment GS has an overall poor prognosis with reported median OS ranging from 8.3 to 16.7 months (5, 25, 29, 37, 43, 53). Compatible with this, median OS for PGS patients in our study was 13.5 months. Although differences in survival between GS and GBM has been reported (5, 39), several studies have failed to reveal any significant difference in OS between GS and conventional GBM (2–4, 25, 29, 37). However, only few studies have investigated difference in survival outcome between GS and conventional GBM patients both receiving trimodality therapy with TMZ-based chemotherapy and these have presented varying results. Damodaran et al. (29) found no significant differences in median OS between a cohort of 12 GS patients and GBM patients receiving treatment according with Stupp's regimen. In a more recent study, Smith et al. (39) compared 14 PGS patients and 256 conventional GBM patients, all treated with TMZ-based chemoradiation, and found a significant worse median OS for PGS patients. In the present study, of nearly twice as many PGS patients, we found no significant difference in median OS when compared to conventional GBM patients (13.4 and 15.7 months, respectively, *p* = 0.201), suggesting that trimodality treatment is just as effective in both groups and consequently supporting continuation of the same clinical management of PGS as conventional GBM.

Our GBM cohort contained 7 SGS patients, all diagnosed at first recurrence of GBM. The current literature on SGS is limited and the pathogenesis of SGS remains unknown (23, 24, 49). Our SGS patients did not present any specific features different from the PGS or GBM patients. Neither did they receive more aggressive treatment than the remaining cohort, which has been proposed as a possible cause for development of these tumors (23). In the largest collective experience with 30 SGS patients, Han et al. (23) reported a median time of 8.5 months from GBM diagnosis to SGS diagnosis and a median OS of 4.4 months from diagnosis of SGS, with a worse outcome among patients who received concurrent and adjuvant TMZ for initial GBM diagnosis. Even though all patients in this study received aggressive RT/TMZ for initial GBM diagnosis, we found compared to this previous study both a longer median time to SGS diagnosis (10.9 months) and median survival from SGS diagnosis (6.6 months). This question the existence of a direct correlation between aggressive treatment and a highly hostile clinical course of SGS patients. Furthermore, with the same survival time from recurrence for PGS and SGS patients, one could speculate if SGS patients represent PGS unrecognized at initial diagnosis due to tumor sampling bias not accounting for extent and heterogenous localization of the sarcomatous presentation in GS.

This study constitutes one of the largest cohorts of GS patients receiving standardized therapy making a regular comparison of treatment outcomes between GS and conventional GBM possible. Although our findings are encouraging, several limitations should be considered, most notably the retrospective study design and the limited statistical power given sample size. The study is disposed to selection bias considering only patients with high pretreatment functional status receive standardized therapy according with the Stupp's regimen. In addition, as the selection for histopathological reevaluation was based upon description of sarcomatous components in the histopathological reports and several histopathological reports contained compendious descriptions, some GS tumors might have gone undiscovered. Regardless, our findings are generally consistent with the existing literature on GS providing an important contribution to the understanding of these rare tumors.

## Conclusion

In this study we found that GS present a similar prognosis as conventional GBM with modern standardized trimodality therapy, indicating that GS may be managed similarly to conventional GBM. Only one PGS patient presented with distant metastasis, suggesting that the incidence of metastases among GS patients may be lower than previously reported. Temporal tumor location and MGMT promoter methylation were significantly associated with survival among PGS patients, supporting the relevance of future studies of RT/TMZ therapy treatment in GS patients to include evaluation of these characteristics.

## Data Availability Statement

All datasets generated for this study are included in the article/Supplementary Material.

## Ethics Statement

The study was conducted in accordance with the Helsinki Declaration and was approved by the Danish National Ethical Committee (1808096). Exemption from obtaining informed consent to participate was granted by the ethical committee as patients were either deceased or fatally ill.

## Author Contributions

SRM, HP, and SM concepted the study. SF and KG evaluated the patient medical journals. SF and HB reevaluated the tissue sections for gliosarcoma diagnosing, while SF and VL evaluated imaging for assessment of tumor location and growth patterns. SF and SRM interpreted the results and drafted the manuscript, with input from HP and SM. All authors reviewed and approved the final manuscript.

### Conflict of Interest

The authors declare that the research was conducted in the absence of any commercial or financial relationships that could be construed as a potential conflict of interest.
