# Supplementary material for: Clinical Characteristics of Gliosarcoma and Outcomes From Standardized Treatment Relative to Conventional Glioblastoma
Source: Front Oncol. 2019 Dec 17;9:1425. doi: 10.3389/fonc.2019.01425 (PMC6928109; doi:10.3389/fonc.2019.01425)
Supplement: Supplementary file 1 [file Table_1.docx]

| **Supplementary Table 1.** Recurrence and salvage therapy. | | | |
| --- | --- | --- | --- |
|  | **GBM**  *(n = 643)* | **PGS**  *(n = 26)* | ***P*-value**  *(PGS vs. GBM)* |
| **Site of recurrence, *n* (%)** |  |  | 0.608 |
| Local | 394 (77.3) | 16 (72.7) |  |
| Distant | 116 (22.7) | 6 (27.3) |  |
| No recurrence | 14 | 1 |  |
| Missing | 119 | 3 |  |
| **Re-resection, *n* (%)** |  |  | 0.408 |
| No | 358 (60.8) | 13 (52.0) |  |
| Yes | 231 (39.2) | 12 (48.0) |  |
| No recurrence | 14 | 1 |  |
| Missing | 40 | 0 |  |
| **Bevacizumab therapy, n (%)** |  |  | 0.840 |
| No | 358 (57.4) | 15 (60.0) |  |
| Yes | 266 (42.6) | 10 (40.0) |  |
| No recurrence | 14 | 1 |  |
| Missing | 5 | 0 |  |
| *Statistical tests:* Fisher’s exact test. *Abbreviations: GBM* glioblastoma, *PGS* primary gliosarcoma. | | | |
