# Supplementary material for: Clinical Characteristics of Gliosarcoma and Outcomes From Standardized Treatment Relative to Conventional Glioblastoma
Source: Front Oncol. 2019 Dec 17;9:1425. doi: 10.3389/fonc.2019.01425 (PMC6928109; doi:10.3389/fonc.2019.01425)
Supplement: Supplementary file 2 [file Table_2.docx]

| **Supplementary Table 2.** Details on MGMT measurements | | |
| --- | --- | --- |
|  | **GBM**  *(n = 643)* | **PGS**  *(n = 26)* |
| **MGMT pyrosequencing, *n* (%)** |  |  |
| Methylated | 89 (48.6) | 3 (25.0) |
| Unmethylated | 94 (51.4) | 9 (75.0) |
| **MGMT IHC, *n* (%)** |  |  |
| Negative | 203 (57.7) | 3 (27.3) |
| Positive | 149 (42.3) | 8 (72.7) |
| Missing | 108 | 3 |
| *Abbreviations: GBM* glioblastoma, *PGS* primary gliosarcoma. | | |
