# Supplementary material for: Clinical Characteristics of Gliosarcoma and Outcomes From Standardized Treatment Relative to Conventional Glioblastoma
Source: Front Oncol. 2019 Dec 17;9:1425. doi: 10.3389/fonc.2019.01425 (PMC6928109; doi:10.3389/fonc.2019.01425)
Supplement: Supplementary file 3 [file Table_3.docx]

| **Supplementary Table 3.** Survival characteristics. | | | | |
| --- | --- | --- | --- | --- |
|  | **GBM**  *(n = 643)* | **PGS**  *(n = 26)* | **SGS**  *(n = 7)* | ***P*-value** |
| **OS from diagnosis (mo), median (range)** | 15.7 (1.1-167.7) | 13.4 (2.3-47.4) | - | 0.201 |
| Missing | 2 | 0 |  |  |
| **PFS from diagnosis (mo), median (range)** | 7.6 (1.1-167.7) | 6.8 (2.3-29.2) | - | 0.105 |
| Missing | 0 | 0 |  |  |
| **Survival from recurrence (mo), median (range)** | 7.4 (0.0-113.7) | 5.8 (0.4-33.6) | 8.6 (7.1-18) | 0.694 |
| No recurrence | 70 | 1 | 0 |  |
| Missing | 2 | 0 | 0 |  |
| *Statistical tests:* Log-rank test.  *Abbreviations: GBM* glioblastoma, *PGS* primary gliosarcoma, *SGS* secondary gliosarcoma, *OS* overall survival, *PFS* progression-free survival. | | | | |
