# Supplementary material for: Clinical Characteristics of Gliosarcoma and Outcomes From Standardized Treatment Relative to Conventional Glioblastoma
Source: Front Oncol. 2019 Dec 17;9:1425. doi: 10.3389/fonc.2019.01425 (PMC6928109; doi:10.3389/fonc.2019.01425)
Supplement: Supplementary file 4 [file Table_4.docx]

| **Supplementary Table 4.** Univariate analyses for OS and PFS in PGS patients. | | | | |
| --- | --- | --- | --- | --- |
|  | **OS** | | **PFS** | |
|  | **HR (95 % CI)** | ***P*-value** | **HR (95 % CI)** | ***P*-value** |
| **Age at diagnosis (per 10 years)** | 0.88 (0.59-1.30) | 0.511 | 0.83 (0.57-1.19) | 0.306 |
| **PS (1-2 *vs*. 0)** | 1.20 (0.53-2.73) | 0.666 | 1.01 (0.44-2.33) | 0.975 |
| **Corticosteroid use (yes *vs*. no)** | 0.84 (0.33-2.15) | 0.713 | 0.61 (0.23-1.63) | 0.322 |
| **Location (temporal *vs.* other)** | 0.41 (0.18-0.94) | 0.036 | 0.45 (0.18-1.10) | 0.079 |
| **MGMT status (methylated *vs.* unmethylated)** | 0.17 (0.04-0.78) | 0.022 | 0.26 (0.07-0.91) | 0.035 |
| **EoR (gross total *vs.* subtotal)** | 1.23 (0.53-2.88) | 0.682 | 0.70 (0.31-1.60) | 0.401 |
| *Statistical tests:* Log-rank test.  *Abbreviations:*  *OS* overall survival, *PFS* progression-free survival, *HR* hazard ratio, *CI* confidence interval, *PS* performance status, *MGMT* O6-methylguanine-DNA-methyltransferase, *EoR* extent of resection, | | | | |
